# The Presence of Rabies Virus-Neutralizing Antibody in Wild Boars (*Sus scrofa*), a Non-Target Bait Vaccine Animal in Korea

**DOI:** 10.3390/vetsci7030090

**Published:** 2020-07-10

**Authors:** Ha-Hyun Kim, Dong-Kun Yang, Ja-Young Wang, Dong-Jun An

**Affiliations:** Viral Disease Research Division, Animal and Plant Quarantine Agency, Gimcheon, Gyeongsangbuk-do 39660, Korea; yangdk@korea.kr (D.-K.Y.); jayoung0256@naver.com (J.-Y.W.); andj67@korea.kr (D.-J.A.)

**Keywords:** neutralizing antibody, rabies virus, wild boars

## Abstract

Oral vaccination with bait is an effective method to prevent rabies in wildlife, but non-target wild animals may also ingest the bait vaccine. In Korea, the target animal of the rabies bait vaccine is the raccoon dog (*Nyctereutes procyonoides*). Bait vaccines have been distributed in Korea for 20 years; although wild raccoon dogs have been tested for antibodies, rabies antibodies have never been investigated in non-target wild animals. Therefore, this study investigated rabies antibody formation in wild boars (*Sus scrofa*), which is likely the main competitor for the bait vaccine in Korea. In bait areas, 20 of 109 wild boars (18.3%) were seropositive, and 39 of 470 wild boars (8.3%) in non-bait areas were also seropositive. These results provide insights regarding bait uptake or vaccination in non-target wild boars.

## 1. Introduction

Vaccines are an important defense and treatment tool in rabies, a deadly zoonosis. Oral vaccine administered using bait is an effective method to prevent rabies in wildlife, such as foxes (*Vulpes vulpes*) and raccoon dogs (*Nyctereutes procyonoides*) [1,2,3]. Vaccines used in bait for oral vaccination consist of modified live rabies vaccines (e.g., Street Alabama Dufferin (SAD) Bern, SAD B19, SAD P5/88, and Street Alabama Gif (SAG)-2), which are mainly used in Europe [4,5], and recombinant vaccines (V-RG, a recombinant vaccinia virus expressing the rabies glycoprotein, and ONRAB^®^, a recombinant human adenovirus expressing the rabies glycoprotein) used frequently in the United States of America (USA) and Canada [6,7]. Bait vaccines have contributed to the eradication of rabies in several European countries, including Switzerland, Estonia, France, Italy, Belgium, and Luxembourg [4,6]. 

In Korea, the raccoon dog has been the main reservoir of rabies in wildlife transmitting rabies to domestic animals, such as cattle, dogs, and cats, since 1993 [8]. The RABORAL V-RG^®^ (Boehringer Ingelheim Animal Health) bait vaccine has been distributed by the Korean Veterinary Authority since 2000 [8]. This bait is constructed of a sachet filled with V-RG and a fishmeal polymer bait cube made of extruded fishmeal and fish oil with a hydrophobic synthetic polymer [6,9]. 

The bait vaccine has been distributed twice annually (March and November) in raccoon dog habitats in Gangwon and Gyeonggi Provinces, as well as in Seoul [8]. Distribution began with 20,000 doses in 2000, with a steady increase in the number of doses up to 990,000 in 2017 [8]. Currently, the annual cost of the bait vaccine is more than 2.5 million dollars. The number of annual rabies cases has gradually decreased from 79 (1 human and 78 animal cases) in 2002 to zero human cases since 2005 and zero animal cases in 2014 [3].

Previous studies in other countries have demonstrated the consumption of the bait vaccine by other wildlife and domestic animals, which consequently produce protective antibodies against rabies virus (RABV) [10,11,12]. Multiple researchers have examined the safety and immunogenicity of V-RG in non-target wild animals [6,13,14]. The safety of V-RG administered via multiple routes was verified in more than 50 warm-blooded vertebrate species including opossum, short-tailed shrews, voles, squirrels monkeys, chimpanzees, ferrets, minks, deer, cattle, sheep, and wild boars [6]. Virus-neutralizing antibody (VNA) titers of ≥0.5 IU/mL were found in common voles (2 of 2, 100%), water voles (4 of 5, 80%), wood mice (16 of 27, 59%), yellow-necked mice (5 of 7, 71%), badgers (2 of 6, 33%), and wild boars (2 of 4, 50%) after direct oral administration of VVTGgRAB-26D3 (or V-RG); none of these animals showed clinical signs [14]. Furthermore, direct oral administration of V-RG did not cause mortality or lesions, and it induced a VNA response to RABV in woodchucks, grey squirrels, coyotes, red-tailed hawks, great horned owls, meadow voles, and gulls [13].

The rabies bait vaccine has been distributed for raccoon dogs in Korea for 20 years and the target animals have been tested for antibody positivity [15,16]. However, rabies antibody positivity, which reflects the rate of bait vaccine consumption, has never been investigated in non-target wild animals in Korea. Therefore, this study investigated the rate of antibody positivity in wild boars (*Sus scrofa*), which are likely the main competitors for the bait vaccine in Korea; it then compared this rate with the reported positive rates in raccoon dogs [16].

## 2. Materials and Methods 

### 2.1. Serum Samples

Wild boars were hunted by the Korean Pork Producers Association and the Korean government for nationwide surveillance according to World Organization for Animal Health (OIE) requirements for classical swine fever (CSF)-free countries. Blood samples of the hunted wild boars were transported to the CSF national laboratory of Animal and Plant Quarantine Agency (APQA). The samples were used for serosurveillance after approval by the APQA, and the Ministry of Agriculture Food and Rural Affairs, Republic of Korea. The CSF national laboratory provided serum samples and information of the wild boars for rabies serosurvey. The rabies bait vaccines were distributed only to Seoul, Gangwon and Gyeonggi provinces, but nationwide samples were used in this study in order to obtain antibody information in bait-vaccinated and non-bait areas.

Among nationwide samples, good quality sera were selected by region to exclude influences of contamination and hemolysis. The serum samples of 579 wild boars hunted from August 2017 to July 2018 (no samples were collected in February 2018) in 10 provinces or cities were examined for VNA titers against RABV. The regions included Gangwon (*n* = 56), Gyeonggi (*n* = 53), Chungbuk (*n* = 125), Chungnam (*n* = 71), Gyeongbuk (*n* = 70), Gyeongnam (*n* = 90), Jeonbuk (*n* = 39), Jeonnam (*n* = 56), Daegu (*n* = 15), and Gwangju (*n* = 4). All serum samples were heat inactivated for 30 min at 56 °C before test.

### 2.2. Fluorescent Antibody Virus Neutralization Test

The RABV VNA of the serum samples was measured according to the OIE Manual with a slight modification [17]. In brief, wild boars serum samples with OIE standard dog serum and negative control dog serum were serially diluted in four consecutive wells of 96-well microplates. Fifty microliters of Challenge Virus Standard (CVS)-11 diluted by 100 tissue culture infectious dose (TCID)_50_/50 μL was added to each well and the microplate was incubated for 1 h in a humid condition with 5% CO_2_ at 37 °C. Then, 50 μL of BHK-21 cell suspension (4 × 10^5^ cells/mL) in Dulbecco’s modified Eagle’s medium (DMEM) supplemented with 10% heat-inactivated fetal bovine serum (FBS) was added and incubated for 48 h in a humid condition with 5% CO_2_ at 37 °C. The cells of microplates were observed by checking cytotoxicity prior to acetone fixation. A few samples showing cytotoxicity were excluded in this study. The cells were washed twice using phosphate buffered saline (PBS) (pH 7.2) and 80% acetone, and were fixed with 80% acetone for 30 min at room temperature. After the cells were dried for 45 min at room temperature, they were reacted with a RABV-specific primary monoclonal antibody (kept in the laboratory) and a fluorescein isothiocyanate (FITC)-conjugated secondary antibody (KPL, Gaithersburg, MD, USA) for 1 h at 37 °C, respectively. The cells were washed three times using PBS after every steps. Cell fluorescence was examined under a fluorescent microscope (Nikon, ECLIPSE, TE2000-U, Tokyo, Japan). Result of each well was read using ‘all or nothing’ method. The titers were calculated with the Spearman–Kärber formula. A RABV VNA titer of ≥ 0.5 IU/mL was taken as the index of the significant protective seroconversion.

### 2.3. Statistical Analysis

All statistical analyses were performed using GraphPad Prism software (Version 8.4.2, San Diego, CA, USA). Fisher’s exact test was used for comparisons between bait-vaccinated and non-bait areas, raccoon dogs and wild boars, and Gangwon and Gyeonggi Provinces. The chi-square test for trend was used for comparisons of seasonal and monthly seropositive rates. A *p* value < 0.05 was considered statistically significant in all test.

## 3. Results

**Figure 1:**
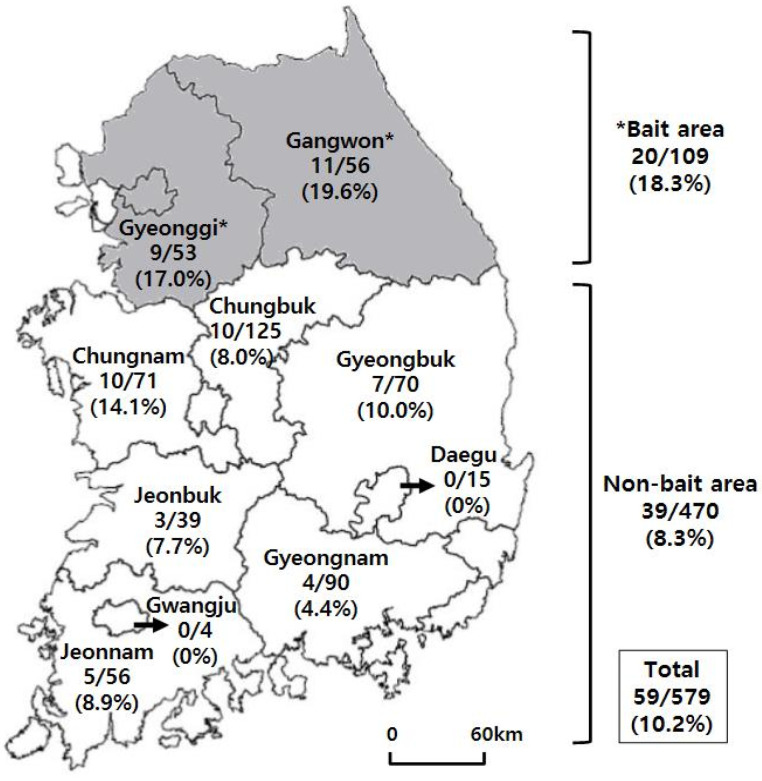
The number of wild boars (No. positive / No. total) and seropositive rate against rabies virus by region. The gray zones indicate the bait-vaccinated area (Seoul, Gangwon and Gyeonggi Provinces). Significant difference was found between seropositive rates of bait-vaccinated and non-bait area (*p* = 0.0042, Fisher’s exact test).

Of the 579 wild boars sampled, 59 (10.2%) were seropositive for RABV VNA (Figure 1). The seropositive rates in the bait-vaccinated areas (Gangwon and Gyeonggi) and non-bait areas (Chungbuk, Chungnam, Gyeongbuk, Gyeongnam, Jeonbuk, Jeonnam, Daegu, and Gwangju) were 18.3% (20/109) and 8.3% (39/470), respectively. The seropositive rate of bait area was significantly different from that of non-bait area (*p* = 0.0042, Fisher’s exact test). When compared by region, the highest positive rate (19.6%, 11/56) was found in Gangwon Province and the next highest (17.0%, 9/53) in Gyeonggi Province. However, no statistical difference was detected between Gangwon and Gyeonggi Provinces, bait-vaccinated areas (*p* = 0.8069, Fisher’s exact test). Seropositive rates greater than 10% were also found in Chungnam and Gyeongbuk, which are adjacent to the two vaccine regions. Four other regions (Jeonnam, Chungbuk, Jeonbuk, and Gyeongnam) had positive rates of 4.4–8.9% (Figure 1). 

Comparing the monthly seropositive rates, the highest positive rates were ≥40% in January and March (no samples were collected in February), followed by a positive rate of 30.8% in May (*p* = 0.0003, chi-square test for trend) (Table 1). Compared with the seasons, it was found that the seropositive rates decreased significantly from spring to winter (*p* < 0.0001, chi-square test for trend) (Table 1).

## 4. Discussion

The wild boar population in Korea has increased steadily, from 3.8 animals per 100 ha in 2012 to an average density of 5.6 animals per 100 ha in 2017 [18]. The densities of wild boars in Gangwon and Gyeonggi, the bait-vaccinated areas, are 2.8 and 6.5 animals per 100 ha, respectively [18]. Wild boars are omnivorous and have the second largest population after water deer (*Hydropotes inermis*) among wild mammals investigated in Korea [18]. In a previous study, 52% of the bait vaccine that disappeared within 72 h after distribution in a restricted area was consumed by wild boar, which find the bait made of a polymer-bound fish meal to be palatable [19]. In field trials, mustelids and wild boars ingested the rabies bait vaccine (SAD B19 vaccine capsule with bait consisting of a mixture of fat, bone, and a fish meal) after distribution [20,21]. Therefore, wild boars could be one of the main non-target animals that compete with raccoon dogs for the consumption of the rabies bait vaccine in Korea.

In mongoose, the seroprevalence of VNAs was high (39.3%) in populations from rabies-enzootic areas without a history of vaccination [22]. Weak VNA titers (0.1–0.2 IU/mL) were detected in 3 of 12 (25%) sham-vaccinated raccoons after RABV challenge at the time of terminal sample collection [23]. In a raccoon-rabies-epizootic area, the seroprevalence of VNAs was 1–3% in the absence of baiting [24]. In an area naïve to baiting but adjacent to baited areas (V-RG), the VNA seroprevalence was 9.6% in wild raccoons (*Procyon lotor*), which are among the bait target animals [25]. Slate et al. (2014) reported that the presence of VNAs before baiting was associated with natural immunity due to sublethal exposure to RABV in raccoon-rabies-enzootic areas, but migration of immunized individuals from the vaccine zone cannot be ruled out entirely as a potential source of the VNAs [25]. To confirm no endemic rabies in any other wildlife spilled from raccoon dogs in Korea, it is necessary to investigate a large number of individuals of various wild animal species. However, the last case of rabies occurred in February 2013, and the brain tissues of 62 dead raccoon dogs in the rabies-outbreak region (Gangwon) were examined in 2016 and 2017; all tissues were negative for RABV [26]. The reduction in the number of rabies cases in the baited area suggests that the probability of RABV exposure is low in the field and that baits are the most likely the source of the detected VNA. Therefore, the higher seroprevalence of VNAs in live wild boars in the baited regions suggests that wild boars consume the bait vaccine distributed in Korea.

In 2012, VNAs against RABV were detected in 20 of 50 (40%) raccoon dog serum samples from Gangwon, Gyeonggi, and Seoul [15]. From January 2017 to June 2018, 13.7% (20/146) of the serum samples from raccoon dogs in Gangwon (17.2% (15/87)) and Gyeonggi (8.5% (5/59)) were rabies-VNA positive [16]. As mentioned above, the prevalence of anti-rabies antibodies has been investigated in the raccoon dog, which is the target wild animal in Korea [15,16]. However, this study is the first to evaluate the prevalence of anti-rabies antibodies in wild boars from multiple Korean provinces. In bait-vaccinated areas of Romania, wild boars, non-target wild animals, exhibited a significantly higher seropositive rate (42.31%, 132/312) than that (28.40%, 98/345) of foxes, which were the target wild animals [12]. In this study, the VNA titer was ≥ 0.5 IU/mL in 59 of 579 (10.2%) wild-boar samples obtained between August 2017 and July 2018 nationwide. In the bait-vaccinated areas of Gangwon and Gyeonggi, 18.3% (20/109) the wild-boar samples were seropositive, and the rate did not differ significantly from recent rates (13.7%, 20/146) reported for raccoon dogs, the target animals (*p* = 0.3845, Fisher’s exact test) [16]. As in the case of raccoon dogs, the seropositive rate in wild boars was also higher in Gangwon (19.6% (11/56)) than in Gyeonggi (17.0% (9/53)), probably because more bait vaccine was distributed in Gangwon than in Gyeonggi [16]. However, no statistical difference was observed between two regions (*p* = 0.8069, Fisher’s exact test). These results suggest that wild boars have ingested bait vaccines and formed antibodies against RABV at levels similar to those in raccoon dogs; moreover, wild boars may reduce the consumption of bait vaccines by raccoon dogs in Korea.

Antibody-positive animals have been detected in non-bait areas as a result of animal migration [10,21]. Antibody-positivity rates of 4.4–14.1% have been detected not only in the regions adjacent to the vaccine areas (Chungnam, Chungbuk, and Gyeongbuk), but also farther away (Jeonnam, Jeonbuk, and Gyeongnam). Wild boars can range over territories of 300–15,000 ha and can migrate up to 100–150 km if high-energy food is insufficient [27]. The extent and purpose of such long-distance movements are unknown, but wild boars in Korea reportedly travel 50–250 km [28]. The wild boar has a lifespan of 8–10 years in the wild and 20 years in captivity [28]. Each V-RG bait contains one single-dose vaccine with a high titer of 10^8.0^ TCID_50_/sachet, and the recombinant vaccine induced long-term protection in wildlife (18 months in adult red foxes) [6]. Therefore, the detection of VNAs in non-bait areas is likely due to movement of wild boars and repeated vaccinations for almost 20 years.

Bait vaccines have been distributed in the rabies outbreak region (Gangwon, Gyeonggi, and Seoul) in March and November each year since 2000. The antibody titer in raccoons induced by oral V-RG peaked after 4–6 weeks and subsequently declined [29,30]. The peak antibody response was observed at 60 days post-vaccination in raccoons vaccinated with ONRAB before challenge [23]. Following oral administration of recombinant rabies virus (ERAGS) to pigs, the antibody level was maintained after 2–4 weeks [31]. Although no study has examined the antibody response over time after oral rabies vaccination in wild boars, the maintaining of antibody levels for 2–4 weeks in pigs could be suggestive for the case in wild boars. Wild boars mate from November to January, and new piglets (litter size 4–10) are born after 4 months, i.e., from late March to early April [28]. Therefore, the seropositive rate (≥ 0.5 IU/mL), after peaking in spring, showed a tendency to decrease due to the birth of wild boars, the death of wild boars (e.g., due to natural causes, capture, and hunting), dispersion, and the post-peak reduction in antibody titer.

## 5. Conclusions

The first investigation of rabies antibody positivity in non-target animals, which might reflect bait vaccine intake, was conducted in Korea, where bait vaccines have been distributed for 20 years. These results indicate that wild boars ingest bait vaccines and produce antibodies against RABV at levels similar to those in raccoon dogs, and that the wild boars may reduce the intake of the bait vaccine by raccoon dogs. RABV-neutralizing antibodies were also detected in animals in non-bait areas, likely due to repeat vaccinations for almost 20 years and to wild boar migration. This information will be useful for evaluating the effect of oral-bait vaccination in wildlife, developing future bait vaccines, and formulating bait-distribution policies. Other wild animal species may also consume the bait vaccine, and thus serosurveillance of other wild animal species in Korea is needed.

## Figures and Tables

**Table 1:** Seasonal and monthly seropositive rates against rabies virus in wild boars (*Sus scrofa*) in Korea from August 2017 to July 2018, with the exception of February (no samples were collected in February 2018).

Season	Seropositivity ^1^ (%) (No. Positive/No. Total Samples)	Month	Seropositivity ^1^ (%) (No. Positive/No. Total Samples)
Spring	24.6 (16/65)	March	41.7 (5/12)
		April	11.1 (3/27)
		May	30.8 (8/26)
Summer	13.5 (7/52)	June	9.1 (2/22)
		July	17.9 (5/28)
		August	0 (0/2)
Fall	8.4 (34/407)	September	1.8 (2/112)
		October	2.0 (2/102)
		November	15.5 (30/193)
Winter	3.6 (2/55)	December	0 (0/51)
		January	50 (2/4)
Total	10.2 (59/579)	Total	10.2 (59/579)
*p* value ^2^	<0.0001	*p* value ^2^	0.0003

^1^ A protective virus-neutralizing antibody (VNA) titer ≥0.5 IU/mL was scored as seropositive against rabies virus (RABV). ^2^ The *p* values were calculated using the chi-square test for trend. A *p* value < 0.05 was considered statistically significant.
